# Analysis of the Cause of Household Carbon Lock-In for Chinese Urban Households

**DOI:** 10.3390/ijerph18042201

**Published:** 2021-02-23

**Authors:** Lingyun Mi, Yuhuan Sun, Lijie Qiao, Tianwen Jia, Yang Yang, Tao Lv

**Affiliations:** School of Economics and Management, China University of Mining and Technology, Xuzhou 221116, China; sunyuhuan@cumt.edu.cn (Y.S.); qiaolijie@cumt.edu.cn (L.Q.); jiatianwen123@cumt.edu.cn (T.J.); yangyang_123@cumt.edu.cn (Y.Y.)

**Keywords:** carbon lock-in, grounded theory, household appliances purchasing, household energy consumption

## Abstract

Household energy conservation is an important contributor to achieve the carbon emission reduction target. However, the actual energy-saving effect of Chinese households is under expectation. One reason for this is because household energy consumption is locked in at a certain level, which has become an obstacle to household carbon emission reduction. In order to reduce this obstacle, this study explored the cause of household carbon lock-in based on grounded theory, targeting newly furnished households. A theoretical model was developed to reveal the formation mechanism of carbon lock-in effect in the purchasing process of household energy-using appliances. NVivo 12 software was used to analyze the decoration diaries of 616 sample households, and the results showed that (1) the direct antecedent of the household carbon lock-in effect was the lock-in of purchasing behavior, and the household carbon lock-in effect was mainly exhibited in the consumption path dependence (of energy-using appliances) and the solidification of energy structure; (2) the willingness to purchase household appliances was the direct antecedent of purchasing behavioral lock-in, and the cost had a moderating effect on the transformation from purchase willingness to behavioral lock-in; and (3) in the process of purchasing household appliances, reference groups, value perception, and ecological awareness can promote purchasing behavioral lock-in by affecting willingness of purchase. Suggestions to promote unlocking of household carbon were also proposed.

## 1. Introduction

Environmental pollution and health threats caused by high carbon emissions are becoming increasingly serious [1,2]. To reduce carbon emissions, China, as the largest carbon emitter in the world, voluntarily pledged at the UN Climate Change Conference in Paris to reduce its carbon emissions by 60–65% of that of 2005 levels by 2030 [3]. Under significant promotion by the government, the carbon emission reduction in the industrial sector has been remarkable [4,5]. However, carbon emissions due to household consumption continues to grow rapidly, accounting for 55.6% of China’s total carbon emissions growth [6]. In addition, Chinese urban households produce 2.89 times more carbon emissions than rural households [7]. Moreover, with the acceleration of urbanization and better electrification of residential households, there is an increasing pressure from household carbon emissions [8,9,10]. Therefore, reducing household carbon emissions plays an important role in achieving China’s energy conservation and emission reduction goals.

The two main factors that determine household carbon emissions are the types and quantities of household appliances, and their usage [11,12]. The impact on the energy consumption of residential buildings caused by physical characteristics of the living environment, including the structure of buildings and the composition of household appliances, is greater than that caused by the occupants’ behavior [13]. Moreover, the types and quantities of appliances purchased by households determine the subsequent scope of usage by the occupants. Occupants’ behavior has a certain degree of embeddedness and dependence on household appliances (that is, user behavior can only be implemented after households have owned household appliances) [14]. When the types and quantities of household appliances in the household are fixed, the energy consumption structure of the household remains stable, which makes the occupants’ energy consumption path fixed, and then the path dependence of energy consumption is produced [15]. This path dependence is essentially, “carbon lock-in” [16].

“Carbon lock-in”, a term originating at the industrial level, refers to the path dependence formed in the coordinated development process of technology and institution, which locks the industrial economy into an energy system based on fossil energy [17]. Similarly, the carbon lock-in effect also exists in household energy consumption due to path dependence. In previous studies, most scholars believed that household carbon lock-in is caused by behavioral lock-in, which is due to individual behavioral habits [16,18]. This conclusion only explains the “dependence” in the “path dependence,” but does not go into how the “path” is formed. In China, the decoration stage of new houses is a critical period for the formation of household energy consumption structures and paths. At present, China’s real estate agencies mostly sell unfurnished housing without decoration, and occupants need to determine the energy-use type in heating and cooling when furnishing a new house (for example, electric heating or water heating, solar energy, or electricity for heating bathing water). At the same time, households also decide the types and quantities of appliances to purchase based on their needs for heating, cooling, cooking, bathing, entertainment, etc. This is the concentrated period for the purchase of household appliances which determines the long-term energy use structure and energy consumption scale of a household for the future. Therefore, taking newly furnished households as examples can better explain the direct cause of the carbon lock-in effect in the purchasing process of appliances in Chinese households. Thus, we considered the households’ decoration diaries as the original data source, which were taken from the “Tubatu” decoration website, one of the largest and most influential online house decoration platforms in China. Then, the performance and cause of the formation of the carbon lock-in effect in the purchasing process of urban household appliances were studied based on grounded theory.

The main contributions of this study are as follows: First, this study shifted the research perspective by focusing on the factors that promoted household carbon emission reduction to barriers. It also expanded the research on carbon lock-in from the industrial level to the micro-household level. Second, this study explored the causes of carbon lock-in in the purchasing process of appliances for urban households in China. It extended research on household carbon lock-in from the use process to the purchasing process, which is the basis for forming carbon lock-in; this is an important innovation that supplements the existing research. Finally, this study confirmed the formation mechanism of carbon lock-in in the purchasing process of household appliances, which provided a foundation for the formulation of carbon unlocking strategies. Our findings also provide a reference for the formulation of policies to promote the use of energy-saving appliances and reduce the total energy consumption of households.

This paper is organized as follows: Section 2 reviews relevant research on household carbon emissions and carbon lock-in. Section 3 summarizes the research methods and data collection process. The analysis process, results, and theoretical model are presented in Section 4. Section 5 details and discusses the theoretical model. Finally, conclusions are presented in the last section.

## 2. Literature Review

### 2.1. Household Carbon Emissions

In recent years, household carbon emissions have been widely discussed and have been a cause for concern [19,20]. The benefits of reducing household carbon emissions for the environment have been proven [21]. In relevant studies, some scholars have tracked the contribution of different household carbon emission sources by measuring household carbon emissions. For example, Cao et al. [22] argued that household appliances and transportation had the highest carbon emission intensity, and that consumption structure and income levels were the two main factors promoting the growth of carbon emissions from households. Ma et al. [23] believed that the air conditioning system, lighting density, and building envelope were the most important factors affecting building energy consumption. Tian et al. [24] found that population size and per capita consumption were the main factors leading to an increase in the household carbon footprint, while carbon intensity had a negative impact on the increase in household carbon footprint. Liu et al. [25] suggested that households with air conditioners or large household appliances were more likely to emit more carbon. In general, the above research suggests that the contributing factors to the increase in household carbon emissions mainly include building characteristics, electrical appliance ownership, household population patterns, and consumption characteristics. These studies suggested mitigation strategies by measuring the main sources of carbon emissions from households. However, there has been no in-depth research on deep-seated psychological motivation for energy consumption leading to the formation of high carbon emission sources in households.

Some scholars have focused on the drivers of household carbon emission reduction from the perspective of residents’ low-carbon behaviors [26]. Among them, internal psychological factors and demographic characteristics were considered as the primary influencing factors [27]. Some scholars have discussed the driving psychology of individual low-carbon behavior based on the theory of planned behavior [28] or value-belief-norm theory [29]. Other scholars have also paid attention to the influence of reference groups [30] and social norms [31]. However, these studies only focused on the psychological factors that drive individuals to engage in low-carbon behaviors, but paid little attention to the barriers that prevent households from reducing carbon emissions. In addition, scholars found that demographic characteristics such as age [32], gender [33], income [34], and education level [35] also affect low-carbon behaviors. However, these factors only reflect the surface differences of low-carbon behaviors and do not explain the underlying causes of their formation. Owing to the consumption path lock-in and structural limitations brought in by household appliances, individual actions to reduce carbon emissions in residential buildings are often less effective than expected [36]. Therefore, the effect of carbon emission reduction at the household level will be greatly reduced if only focusing on how to promote low carbon emissions and ignoring carbon lock-in, which is the obstacle to emission reduction.

### 2.2. “Carbon Lock-In” in Household Energy Consumption

The term “carbon lock-in” was first proposed by Unruh [17], who pointed out that the industrial economy is locked into an energy system based on fossil energy because of increasing returns to scale and constraints on technology and institutions. The techno-institutional complex is a carbon lock-in. This definition has been widely used to explore the effect of industry carbon lock-in at the macro level [37]. In recent years, few scholars have focused on household carbon lock-ins. For example, Christensen [14] found that lifestyle would have a significant impact on household energy consumption, and a lifestyle with high consumption would cause a large amount of energy to be locked in to daily life. Maréchal [18] believed that deep-rooted habits affect people’s energy consumption behavior and make people perform behavioral lock-in; these habits are formed by repeated activities in a stable environment [18]. Therefore, a stable environment is a prerequisite for using behavioral lock-ins. In household energy consumption, a stable environment is established from the time the appliances are purchased. Although the ownership of appliances does not directly affect energy consumption, the types and quantities of appliances purchased and owned by a household determine its basic level of energy consumption [38]. For example, if a household buys a high-power appliance, it will have the opportunity to use it. Once the appliance is turned on, it consumes more energy than a low-power appliance with the same function in the same amount of time. This keeps household energy consumption and carbon emissions fixed at a certain level that cannot be reduced. This also explains why some scholars have found that ownership of some large appliances can lead to a higher level of carbon emissions [25]. Therefore, this study goes beyond previous studies that mainly focus on using behavioral lock-in and traces the pre-cause of household carbon lock-in from the source. The study explores why household energy consumption cannot be reduced from the purchasing process of household appliances.

Although scholars have paid attention to the existence of the lock-in effect in household carbon emissions, existing studies mainly focus on the behavioral lock-in in the use process and ignore the carbon lock-in in the purchasing process. However, as the precondition for the occurrence of user behavior, purchasing behavior is equivalent to the “threshold” of it, which plays an important role in the whole process of energy consumption. Therefore, this study focuses on the carbon lock-in effect during the purchasing process.

## 3. Methods and Data Collection

### 3.1. Methods

**Figure 1:**
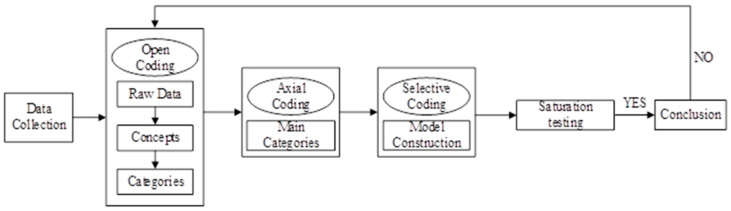
Process of grounded theory.

The data used in this study were from the decoration diaries recorded by the owners on the home decoration website [39]. The purpose of this study was to summarize the theoretical framework of carbon lock-in, which is formed in the purchasing process of urban household appliances. Therefore, it was appropriate to use the qualitative research method of grounded theory. Grounded theory was first formally proposed by Glaser and Strauss in 1967 [40]. They defined the theory as “The discovery of theory from data—systematically obtained and analyzed in social research” [41]. This theory provides a set of strategies for rigorous qualitative research [42]. When using grounded theory for research, there are generally no theoretical assumptions as prerequisites. The original empirical data collected from direct observations was summarized, and the relevant core categories were extracted. Through the analysis of correlations among categories, a theoretical model was eventually established. The specific steps can be divided into three stages: data collection, three-layer coding (open coding, axial coding, and selective coding), and theoretical saturation test [43]. The grounded theory process is shown in Figure 1.

### 3.2. Data Collection

The original data for the grounded theory used in this study were all collected from the owner diaries on the official website of Tubatu. Tubatu is one of the largest online household decoration platforms in China, with branches in 328 cities. It provides a one-stop household decoration service for users, serving more than 29 million Chinese families. Tubatu encourages house owners to keep decoration diaries to share their decoration experience, comment on the decoration company, and the list of items purchased during the decoration process on the decoration diary column of its official website. The purpose of this column is to provide a reference for other house owners who have intentions to decorate.

As of 31 December 2019, there were 3813 owners recording decoration diaries on the Tubatu website, and the number of diaries recorded by each owner was more than one. Considering the integrity and richness of information, we set certain screening conditions for the selection of the owners: (1) the owner kept more than 30 decoration diaries; (2) the owners’ diaries record the purchase lists of household appliances. After screening and sorting, 720 owners were selected. Subsequently, we removed 104 owners with incomplete household appliance information and finally obtained the decoration diaries of 616 households. The sample structure characteristics of the 616 households are listed in Table 1.

There were three main categories in the data, including basic information, sentence data, and information about household appliances. Basic information was expressed by the owners’ registration ID, gender, decoration time, city, residential area, etc. Sentence data were excerpts from the owners’ diaries. To ensure the objectivity of the data, the original state of the sentences was maintained when extracting them. Information on household appliances was mainly collected from the owners’ shopping lists, which included the name, type, unit price, power, the quantity of each type, and the total number of appliances.

## 4. Results

### 4.1. Analysis of Energy Consumption Structure and Types of Household Appliances

**Figure 2:**
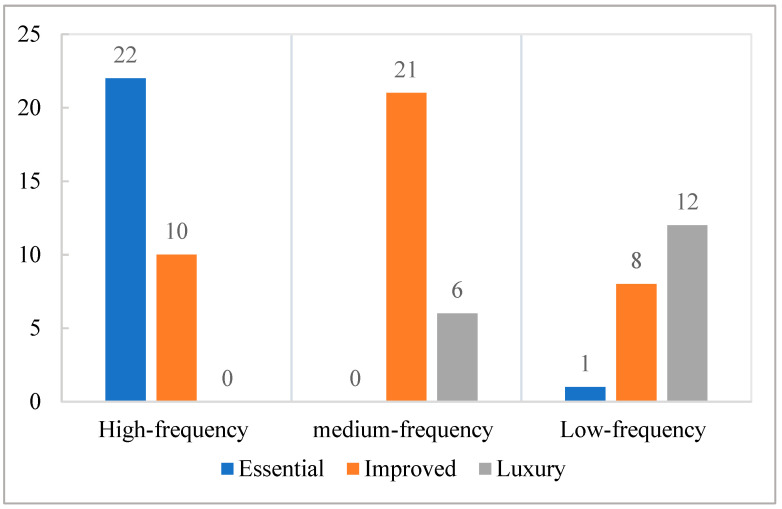
Type distribution of household appliances.

In this study, we conducted a statistical analysis of the types and purchasing proportions of appliances purchased by 616 households to understand the energy consumption structure of newly furnished households and their consumption path structures. Statistics showed that there were 80 kinds of appliances recorded on the shopping lists of 616 households. According to the number of households purchasing a certain appliance, the appliances were divided into high-proportion, intermediate-proportion, and low-proportion appliances, with a purchasing proportion of over 50%, between 10% and 50%, and less than 10%, respectively. In addition, according to the dependence on these appliances in modern life (the level of substitutability), we divided household appliances into three categories: basic appliances (necessary appliances in daily life, such as televisions, washing machines, refrigerators, air conditioners, lamps, and conventional bathing equipment), improved appliances (appliances that can be replaced manually or with non-energy consuming tools, such as electric mops and floor mopping robots), and luxury appliances (appliances beyond the scope of people’s daily needs, which have a certain symbolism, functionality, and experience [44], such as high-end home theaters). The specific classification of household appliances is listed in Table 2. Figure 2 shows the type distribution of the household appliances.

As shown in Table 2, most of the appliances in newly furnished households consume electricity, and only a small number of appliances consume natural gas or solar energy. Natural gas and solar energy as renewable energy can not only reduce carbon emissions but also reduce usage costs [45]. However, clean energy accounts for a relatively low proportion, while electricity accounts for a higher proportion in newly furnished households. In China, 70.41% electricity depends on coal conversion, leading to higher carbon emissions from electricity use [46,47]. Therefore, the characteristics of the energy-using structure in the households have brought about the solidification of the carbon emission structure, which maintains the household carbon emissions at a high level.

In addition, classified statistics based on the degree of necessity and purchasing proportion reflect the changing characteristics of the types and quantities of household appliances. This change determines the characteristics of household consumption path dependence. The results in Table 2 and Figure 2 show that the number of basic appliances bought by newly furnished households is increasing. For example, in the installation of lamps, besides those satisfying basic lighting demands, many households will install additional decorative lamps. Moreover, an increasing number of households are buying improved appliances, such as dishwashers, improved energy-using appliances, and high-proportion energy-using appliances. Meanwhile, the purchasing proportion and types of luxury appliances for newly furnished households, such as treadmill, which is a luxury energy-using appliance and an intermediate-proportion energy-using appliance, are constantly increasing.

Additionally, with the improvement of living standards, residents’ have not been satisfied with basic appliances. To make life more convenient, healthy, and interesting, residents’ demands for improved and luxury appliances are continuously increasing, which has increased the number and structural upgrading of appliances in newly furnished households. Such characteristics of appliance ownership will lead to a fixed energy consumption path in the future, and will result in the formation of household carbon lock-in. However, the specific reason for this formation remains to be further studied based on the grounded theory.

### 4.2. Analysis of the Cause of Carbon Lock-In Based on the Grounded Theory

In this study, the three-level coding process of grounded theory was completed using NVivo 12. First, the original statements from the owners’ diaries were placed in order. Then, files containing the sorted statements were imported into NVivo 12. The data need to be processed during data collection if the number of samples studied is unknown [48]. The sample size of this study was fixed; therefore, we encoded them uniformly after data collection. The collected raw data were divided into two groups for comparative analysis to test whether there were any new categories [49]. Therefore, we randomly selected diaries of 410 owners (2/3) for analysis, and another 206 samples (1/3) were used for the test of new categories.

#### 4.2.1. Open Coding

In the coding process of grounded theory, the first is open coding or level 1 login. Open coding consisted of analyzing and sorting the raw data of the owner’s diaries word by word, and then generating concepts and discovering categories [50]. The qualitative analysis software NVivo 12 was used for the open coding process of the original text data in the owners’ diaries, following the coding process of “raw data→labeling→conceptualization→categorization”. First, we randomly allocated the original sentence data of 410 owners’ diaries in sequence, imported them into NVivo 12 individually, and labeled the original data. In total, 2370 reference points were created. Then, the reference points were conceptualized according to the standards that they are fit and related. There are many initial reference points, but many of them are crossed and repeated. Therefore, we chose those with more than 5 repetitions and eliminated those single contradictory initial concepts, and a total of 30 concepts were obtained. Finally, 11 categories were formed through further screening and merging of the concepts. Table 3 shows the results of open coding. Restricted by the length, one sentence was selected as a representative of each concept in Table 3.

#### 4.2.2. Axial Coding

Open coding is followed by axial coding, which is also known as associative login or axis login. The purpose of axial coding was to explore the relationship between the independent categories formed in the open coding stage, discover the commonalities and logical connections between the categories, and form a clearer and more precise main category [51,52]. In the axial coding phase, the relationships between the 11 categories obtained in the open coding phase were refined and classified. Finally, 5 main categories were identified. The results of the axial coding and the definitions of each category are listed in Table 4. 

#### 4.2.3. Selective Coding and Model Construction

Based on the completion of the axial coding, selective coding was carried out. This step is also known as core login, which lays the foundation for the construction of the theory. Selective coding is used to explore the core category from the main categories and build a typical relationship structure between the main categories around the core category [53]. Then, through the analysis of the logical relationship, each category system is integrated into the same storyline, and the relationship is verified to build a theoretical model. In the axial coding stage, five main categories, namely carbon lock-in phenomenon, reference groups, value perception, ecological awareness, and cost, were formed. In the selective coding stage, we further clarified the relationship between these five categories, established a relationship structure with “influencing mechanism of purchasing behavioral lock-in” as the core category, and formed a theoretical model.

**Figure 3:**
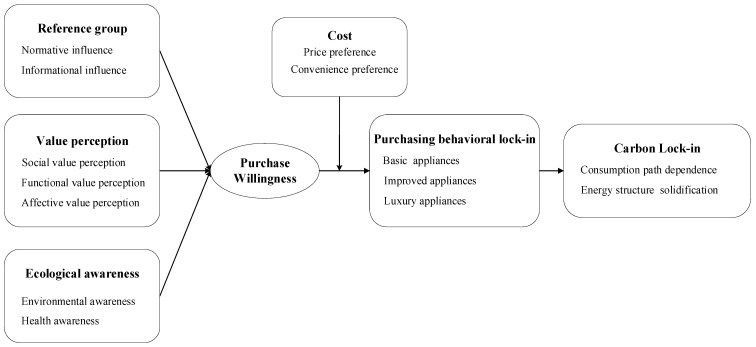
Theoretical model of the formation mechanism of carbon lock-in effect in the purchasing process of household appliances.

The storyline around the core category can be described as follows: Purchasing behavioral lock-in leads to the purchase of a large number of basic appliances. The purchasing proportion of improved and luxury appliances is high, and many types of luxury appliances are purchased. These purchase characteristics cause the solidification of the energy structure and dependence of the consumption path, which leads to carbon lock-in. Purchasing behavioral lock-in is affected by purchase willingness, and the above relationship is moderated by cost. Meanwhile, the purchase willingness of appliances is affected by the reference groups, value perception, and ecological awareness. Figure 3 shows the theoretical model of the formation mechanism of the carbon lock-in effect in the purchasing process of household appliances.

### 4.3. Theoretical Saturation Test

Based on the theoretical saturation test proposed by Pandit [52], this study introduced 206 reserved owner diaries into NVivo 12 for a new round of three-layer coding. The results showed that there were no new concepts, categories, and relationships that were significantly different from the previous study, indicating that the above theoretical model was saturated.

## 5. Model Interpretation and Discussion

At present, research on carbon lock-in mainly focuses on the industrial level, but only a few studies focus on the household level. Research on household carbon lock-in emphasizes the use process of appliances. However, the essence of carbon lock-in is path dependence, and the path is formed during the purchasing process of household appliances. Therefore, it is more meaningful and interesting to study the household carbon lock-in mechanism from the perspective of the purchasing process of appliances. For that purpose, this study considered households with newly furnished houses as examples, using the owners’ decoration diaries collected from Tubatu decoration website as the data source, and established the model of carbon lock-in effect mechanism in the purchasing process of household appliances based on grounded theory. This study explored the cause of household carbon lock-in and provided a reference for proposing strategies for household carbon unlocking. The main findings of this study are discussed below.

(1) The energy consumption path dependence and the consumption structure solidification caused by the purchasing behavioral lock-in of household appliances were direct reasons for the formation of household carbon lock-in. Household purchasing behavioral lock-in refers to the fact that residents form certain purchasing habits due to the influence of some factors, and have a fixed selection tendency when purchasing household appliances [54,55]. Our research shows that most households currently have similar purchasing behavioral lock-in characteristics. For example, household appliances are mainly electrical appliances, and the number of basic appliances purchased is large. The number of households that bought improved and luxury appliances increased, and the types of luxury appliances also increased. Such characteristics make household energy consumption paths and consumption structures more stable. In this stable environment, the high energy consumption behavior of residents leads to carbon lock-in [18]. Our results were similar to the findings of Attari et al. [56], who also found that the choice of purchasing patterns has an important impact on household carbon lock-in. Therefore, changing the purchase patterns is beneficial for reducing household energy consumption. In summary, government departments should focus on promoting the development of the new energy industry, such as promoting technological innovation in the field of new energy. At the same time, attention should be paid to improving the development efficiency of photovoltaic and wind energy industries, promoting the development of green and sustainable energy (e.g., nuclear, hydrogen, and bioenergy), and constantly increasing the proportion of green energy in household energy consumption structure.
(2) The willingness to purchase household appliances is the direct antecedent of purchasing behavioral lock-in, and the cost plays a moderating role in transforming the willingness to purchase behavior. Willingness is considered to have the most direct predictive effect on behavior [57,58,59]. Similarly, consumers’ willingness to purchase is also a direct driver of actual purchasing behavior. However, it is worth noting that there is a gap between willingness and behavior; that is, not all willingness is converted into behavior [60,61]. An online experiment conducted by Farjam et al. [62] on 660 adults in the United States showed that cost can reduce the environmental attitude–behavior gap. This study also found that cost is one of the reasons for the willingness–behavior gap. The higher the cost, the lower the possibility for willingness to transform to behavior. When consumers hesitate between two energy-using products with similar performance, they will choose products with a lower price or that which is easier to transport. Thus, consumers may give up purchasing energy-using products when they find it difficult to purchase or transport, or because of the expensive price. Therefore, cost is an important factor in changing purchasing behavioral lock-in, which should be considered when formulating the household carbon unlocking policy. First, government departments can increase subsidies to households purchasing green energy products. For example, manufacturers are encouraged to provide home delivery and extended warranty services to residents who purchase green appliances. Second, the government could improve the product carbon labeling system, and implement corresponding rewards and penalties for households buying electrical appliances based on carbon labels. Last, sellers should inform buyers of the energy consumption and carbon emissions of various appliances at the time of purchase. For example, an air conditioner consumes 291.06 TWh/year and emits 950.69 kg CO_2_ eq./year, a desktop computer consumes 88.30 TWh/year and emits 607.79 kg CO_2_ eq./year, a refrigerator consumes 84.64 TWh/year and emits 253.00 kg CO_2_ eq./year, and a TV consumes 113.63 TWh/year and emits 221.19 kg CO_2_ eq./year [63]. In addition, through the carbon tax or household-level carbon trading to increase the cost of high-carbon appliances, and encourage residents to choose lower-carbon products.
(3) Reference groups, value perception, and ecological awareness can promote purchasing behavioral lock-in by affecting willingness of purchase. The influence of the reference groups mainly includes normative and informational influences. Some scholars believe that reference groups will have an impact on non-green consumption behavior [64]. Studies have also shown that consumers’ decisions are often influenced by neighbors, colleagues, opinion leaders, and other peers [65]. On the one hand, the information provided by reference groups affects the individuals’ willingness to buy because it affects consumers’ expectations of product performance and thus affects their preferences [66]. Therefore, households that are susceptible to informational influence may not be able to cope with the salesperson’s vigorous promotion and purchase energy-using products that are not necessary in the plan. They may also be encouraged by a decoration company or by netizens to buy superfluous energy-using products. On the other hand, households are susceptible to pressure from surrounding groups to change their consumption behaviors to meet the preferences, standards, and norms of reference groups [30]. Therefore, normative impact is also an important factor affecting the willingness of residents to purchase appliances.

Value perception is a factor that directly affects the willingness of households to purchase appliances, including social value, functional value, and affective value perception. Households that are sensitive to the social values may purchase more high-tech or high-power energy-consuming products because of reasons such as the desire to show their social status. Households that are more sensitive to the functional values may be willing to purchase energy-using products with complete functions, more stable performance, or more mature technologies. These products can bring excellent user experience to consumers and affect their willingness to purchase [67]. They may also prefer to buy energy-using products with combined functions because they are not satisfied with a single function. Those who are sensitive to the affective values are more willing to purchase energy-using products with higher comfort, better service, or product experience. After the basic energy-using products sufficiently meet the demands of their households, they may buy more energy-intensive products to make their lives better and more comfortable. Therefore, the government can increase the publicity of negative environmental information, so that the public can be aware of the grim situation of the current environment. At the same time, it should strengthen environmental protection education to the public, advocate low-carbon consumption values, and weaken the influence of the value of material hedonism.

The influence of ecological awareness is another psychosocial factor that directly affects the willingness to purchase. Households’ concerns about climate change, air quality, and health status, as well as their energy-saving and environmental protection willingness are examples of ecological awareness. Households that are more concerned about health would pay close attention to climate change and air pollution. However, they tend to buy energy-using products that make them healthier and reduce the damage of the outside environment to the health, such as buying air purifiers, fresh air systems, and treadmills. The purchase of air purifiers by residents out of health awareness helps to improve the air quality in their houses, but it increases the carbon emissions to the external environment, which is not conducive to the improvement of the overall environment. Therefore, how to coordinate the conflict between the private and public environmental protection interests becomes a problem worthy of attention. Policymakers need to find a win-win path in the environmental protection needs for all parties, so that residents’ health awareness can truly be transformed into the improvement of the social environment. Households with higher energy savings and environmental consciousness may buy energy-efficient products. Some scholars believe that consumers who are concerned about environmental issues are more willing to buy environmentally friendly products and are willing to pay higher prices for these products [68]. Additionally, households with higher environmental awareness tend to purchase more energy-saving equipment [69]. However, similar to the rebound effect, although the energy consumption per product is reduced, the increase in the types and quantities of appliances may lead to an increase in the total energy consumption.

## 6. Conclusions

Grounded theory was adopted to develop the carbon lock-in model of household energy-using appliances in the purchasing process. The model showed that the purchasing behavioral lock-in lead to path dependence and structural solidification of household energy consumption and, furthermore, created the carbon lock-in effect. In addition, the households’ willingness to purchase appliances can directly influence the purchasing behavioral lock-in. Meanwhile, the influencing mechanism can be adjusted by cost. The willingness to purchase appliances was also affected by reference groups, value perception, and ecological awareness. The findings can guide the government to intervene in the behavioral lock-in in the purchasing process and to plan for unlocking measures.

To the authors’ knowledge, this article is one of the few papers focusing on carbon lock-in during the purchasing process of household appliances. This is also the first study on the carbon lock-in effect of newly decorated households in China. This study provides a new perspective for studying carbon emission reduction in households and enriches the theoretical basis for relevant research. Although this research certainly has academic and practical contributions, it also has several limitations. First, our sample was mainly concentrated in China’s first- and second-tier cities. In future research, the scope of data collection will have to expand to other cities with different levels of development. Second, this study uses only a qualitative research method based on grounded theory to establish a theoretical model. The model was validated using questionnaires or experiments. Finally, this study focuses on the carbon lock-in caused by the purchase of electrical appliances in newly furnished households. However, the impact of other factors on household carbon lock-in, such as the structure of buildings before renovation [70] (e.g., wall and ceiling insulation, natural ventilation and shading) and energy consumption behavior, household size, and other sociodemographic attributes after the residence has not been considered. It can be further explored in future research.

## Figures and Tables

**Table 1:** Description of sample structure characteristics.

Variable	Category	No.	Percentage	Variable	Category	No.	Percentage
Gender	Male	147	23.86%	Residential area	60 m^2^ and below	94	15.26%
	Female	469	76.14%		61–80 m^2^	97	15.75%
City	First-tier	234	37.99%		81–100 m^2^	116	18.83%
	Second-tier	288	46.75%		101–120 m^2^	101	16.40%
	Third-tier and below	94	15.26%		121–150 m^2^	126	20.45%
					151 m^2^ and above	82	13.31%

**Table 2:** Classification of household appliances.

Type	Appliances
High-proportion appliances	Television, refrigerator, range hood, gas stove, microwave oven, rice cooker, induction cooker, electric kettle, soymilk maker, electric water heater, bathroom heater, split air conditioner, ventilation fan, router, computer, printer, washing machine, hair dryer, lighting fixture, water dispenser, heater, electric pressure cooker, decorative lamp, liquidizer, oven, humidifier, dishwasher, vacuum cleaner, instant heating type kitchen Po, garment steamer, gas water heater, and smart disinfection cabinet
Intermediate-proportion appliances	Egg steamer, integrated stove, electric cooker, electric hot pot, food processer, electric steaming box, gas-fueled floor heating system, electric oil heater, electric fan, electric faucet, air purifier, intelligent closestool, floor mopping robot, electric mop, electric drying rack, garbage disposer, central ventilation system, treadmill, dryer, monitoring system, gas fireplace, food mixer, mite remover, toaster, high-end stereo system, and wall breaking machine
Low-proportion appliances	Electric sewing machine, coffee machine, electricity-powered floor heating system, solar water heater, feet warmer, electric iron, steam mop, central air-conditioning system, dehumidifier, high-end home theater, large game console, projector, face steaming device, pet dryer, surf jacuzzi, hand dryer, massage chair, water tank thermostat, mahjong machine, gramophone, fruit and vegetable detoxifying machine, and water purifier
Basic appliances	Television, refrigerator, range hood, gas stove, microwave oven, rice cooker, induction cooker, electric kettle, soymilk maker, electric water heater, bathroom heater, split air conditioner, ventilation fan, router, computer, printer, washing machine, hair dryer, lighting fixture, water dispenser, heater, electric pressure cooker, and electric iron
Improved appliances	Decorative lamp, integrated stove, liquidizer, oven, humidifier, dishwasher, vacuum cleaner, garment steamer, egg steamer, smart disinfection cabinet, electric cooker, electric hot pot, food processer, electric steaming box, gas-fueled floor heating system, electric oil heater, gas water heater, electric fan, electric faucet, instant heating type kitchen po, air purifier, intelligent closestool, floor mopping robot, electric mop, electric drying rack, electric sewing machine, toaster, coffee machine, food mixer, electricity-powered floor heating system, solar water heater, feet warmer, steam mop, gas fireplace, projector, gramophone, high-end stereo system, wall breaking machine, and water purifier
Luxury appliances	Garbage disposer, central ventilation system, treadmill, dryer, monitoring system, central air-conditioning system, dehumidifier, high-end home theater, large game console, face steaming device, mite remover, pet dryer, surf jacuzzi, hand dryer, massage chair, fruit and vegetable detoxifying machine, water tank thermostat, and mahjong machine

**Table 3:** Results of open coding.

Categories	Representative Sentence (Concepts)
Energy structure solidification	I bought a lot of new electrical appliances for this renovation, which mainly use electricity every day. (Electricity consumption solidification)
	I used a solar water heater before, but it is not stable. This time, I decide to use a gas water heater instead. (Give up the green energy)
Consumption path dependence	In this renovation, I bought new appliances such as electric steaming box, dishwasher, intelligent closestool, and so on to modernize my daily life. (Life style)
	I am used to drinking soybean milk and porridge every morning, so I bought a wall breaking machine. (Living habit)
Normative impact	I want to buy a gas water heater, but my parents asked me to buy an electric water heater, so I finally chose the electric one. (Influence of relatives)
	My neighbors told me that they all use the range hood of this brand, so I bought one. (Influence of neighbors)
	A friend recommended me to buy this treadmill. After I tried it, it was really good and I placed an order. (Influence of friends)
	My colleague called me to visit the dishwasher of FOTILE. It felt good and I ordered it immediately. (Influence of colleagues)
Informational impact	Originally, I wanted to buy a refrigerator of Midea, but after being persuaded by the salesmen I decided to buy a Siemens one (Influence of salesperson)
	I found the electric kettle on the Internet. The comments of netizens are generally good, so I finally decided to buy it. (Influence of netizens)
	This TV is recommended by the decoration company. I think it’s very good. (Influence of decoration companies)
Social value perception	We finally decided to buy the vertical air conditioner, which looks very upscale in the living room. Guests may admire it when they come. (Face consciousness)
	It’s safe to use such a first-line brand as FOTILE! The quality of their products is very reliable. (Brand)
	The appearance of the electrical appliances should be consistent with the decoration style of my house, otherwise, they are not beautiful enough. (Appearance)
Functional value perception	I want to buy a Rinnai water heater because I had one before. It is of good quality and we had been using it for many years. (Quality)
	Safety should be considered when buying water heaters firstly. I feel that electric water heaters are safer than gas water heaters, so I bought an electric one. (Safety)
	When choosing appliances, I will value the product’s high-cost performance. (Product economy)
	I bought an air conditioner with a formaldehyde removal function, which is good for my health. (Function)
	I decided not to buy the embedded microwave oven, just to buy a common one. I think practicality comes first. (Practicability)
Affective value perception	The heating equipment is being installed today. A.O. Smith’s service is really good. I love it! (Service experience)
	I finally brought a massage chair. Although its price is very high, using a massage chair after work will make me feel very comfortable. (Comfort preference)
Environmental awareness	The circulation system of this gas water heater monitors the heating system in real-time, and the energy-saving effect is very significant. (Energy conservation)
	The food waste processor can smash food waste and flush it down the drain to reduce food waste. (Reducing pollution emissions)
Health awareness	I bought a smart disinfection cabinet, water purifier, and fruit and vegetable detoxifying machine to ensure safer food. (Promoting human health)
	The smog pollution is serious now, so the whole house is decorated with a central ventilation system. (Reduce harm to the body)
Price preference	I bought a Siemens washing machine during Double Eleven and it was very cheap. (Cheap to buy)
	I bought a Midea air conditioner and heard that it’s “one kilowatt per night”, so I don’t have to worry about the electricity bill when I turn on the air conditioner. (Low cost of use)
Convenience preference	There are often activities on JD’s electrical appliances, and it is particularly convenient to purchase through the mobile APP. (Convenient to buy)
	Now when you buy electrical appliances, the merchants will be responsible for the delivery and installation, saving a lot of trouble. (Convenient installation and transportation)
	I changed the electric water heater into a gas water heater so that I can burn water and take a bath without waiting. It’s very convenient. (Convenient to use)

**Table 4:** Results of axial coding.

Main Categories	Categories	Definition
Carbon lock-in phenomenon	Energy structure solidification	The energy type of household energy consumption has been fixed in the future.
	Consumption path dependence	The stability of energy consumption path brought about by the dependence of household daily life habits on electrical appliances.
Reference groups	Normative impact	It refers to the impact that an individual is influenced by the people around him/her and wants to be consistent with them.
	Informational impact	The influence of information about the specific function and performance of products on the willingness of individuals to purchase.
Value perception	Social value perception	The social satisfaction an individual gets from using products or services.
	Functional value perception	The basic use-value of a product or service. It is an individual’s perception of products or services in terms of functionality, practicality, and use performance.
	Affective value perception	The feelings or emotions such as happiness, relaxation, and excitement that are produced in the process of using products or services.
Ecological awareness	Environmental awareness	Individual’s concern for the environment and awareness of energy conservation and emission reduction.
	Health awareness	Individuals’ awareness of the benefits and harm of appliances to human health.
Cost	Price preference	Individuals’ preference for low prices in the process of buying or using products.
	Convenience preference	Individual’s preference for convenience in the process of purchasing, installing, transporting, and using the product.

## Data Availability

The data that support the findings of this study are available from the corresponding author, upon reasonable request.
